# Update on the NCEP ATP-III emerging cardiometabolic risk factors

**DOI:** 10.1186/1741-7015-12-115

**Published:** 2014-08-26

**Authors:** Robert H Eckel, Marc-Andre Cornier

**Affiliations:** Division of Endocrinology, Metabolism and Diabetes, University of Colorado School of Medicine, Anschutz Medical Campus, Mail Stop 8106, 12801 E 17th Ave, Aurora, CO 80045 USA

**Keywords:** Apolipoprotein B, Lipoprotein (a), C-reactive protein, Homocysteine

## Abstract

The intent of this review is to update the science of emerging cardiometabolic risk factors that were listed in the *National Cholesterol Education Program (NCEP) Adult Treatment Panel-III* (ATP-III) report of 2001 (updated in 2004). At the time these guidelines were published, the evidence was felt to be insufficient to recommend these risk factors for routine screening of cardiovascular disease risk. However, the panel felt that prudent use of these biomarkers for patients at intermediate risk of a major cardiovascular event over the subsequent 10 years might help identify patients who needed more aggressive low density lipoprotein (LDL) or non-high density lipoprotein (HDL) cholesterol lowering therapy. While a number of other emerging risk factors have been identified, this review will be limited to assessing the data and recommendations for the use of apolipoprotein B, lipoprotein (a), homocysteine, pro-thrombotic factors, inflammatory factors, impaired glucose metabolism, and measures of subclinical atherosclerotic cardiovascular disease for further cardiovascular disease risk stratification.

## Introduction

It has been long known that certain factors and conditions are associated with increased risk for cardiovascular disease (CVD) and when present warrant more aggressive management. These major risk factors include age, sex, family history, hypertension, diabetes, cholesterol and smoking, with elevated high density lipoprotein (HDL) cholesterol as being protective or a ‘negative’ risk factor. These major risk factors were the basis for the recommendations set forth by the National Cholesterol Education Program (NCEP) Adult Treatment Panel-III (ATP-III) report of 2001 [1] (updated in 2004) [2]. A number of other cardiometabolic risk factors, so called ‘emerging risk factors,’ have also been identified and reviewed [3, 4]. These risk factors include, but are not limited to, obesity, metabolic syndrome, hypertriglyceridemia, apolipoprotein B, lipoprotein (a), homocysteine, pro-thrombotic factors, pro-inflammatory factors as well as measures of subclinical atherosclerotic cardiovascular disease (ASCVD). At the time the ATP-III report was published the evidence was felt to be insufficient to recommend these risk factors for routine screening of CVD. However, the ATP-III panel felt that prudent use of these biomarkers for patients at intermediate risk of a major CVD event over the subsequent 10 years might help identify patients who needed more aggressive low density lipoprotein (LDL) or non-HDL cholesterol lowering therapy.

The more recent 2013 American College of Cardiology/American Heart Association (ACC/AHA) Guideline on the Assessment of Cardiovascular Risk has also made recommendations on the use of some of these emerging risk factors, including markers of inflammation and subclinical ASCVD [5]. The European (European Guidelines on Cardiovascular Disease Prevention in Clinical Practice) [6] and Canadian (2012 Update of the Canadian Cardiovascular Society Guidelines for the Diagnosis and Treatment of Dyslipidemia for the Prevention of Cardiovascular Disease in the Adult) [7] guidelines have also been recently updated, both reviewing and making recommendations on a number of these emerging cardiometabolic risk factors. These recommendations have been summarized in Table 1 illustrating the lack of consensus regarding these risk factors.

**Table 1:** **European, Canadian and ACC/AHA guidelines on the use of emerging risk factors**

Emerging risk factor	European [6]	Canadian [7]	ACC/AHA [5]
**Apo B**	No added value; may be a more accurate assessment of CVD risk versus LDL-C in patients with hypertriglyceridemia	≥120 mg/dL as an alternative marker for intermediate risk patients with LDL-C <3.5 mmol/L	Screening not recommended
**Lipoprotein (a)**	Screening not recommended	Consider for intermediate risk patients. Levels >30 mg/dL considered higher CVD risk	Screening not recommended
**Homocysteine**	May be used in persons at moderate CVD risk.	Screening not recommended	Screening not recommended
**Pro-thrombotic factors**	Fibrinogen may be used in persons at moderate CVD risk.	Screening not recommended	Screening not recommended
**Pro-inflammatory factors**	hsCRP may be used in persons at moderate CVD risk.	Screening not recommended	Consider screening with hsCRP for intermediate risk patients and consider statin therapy for patients with levels ≥2 mg/dL.
**Impaired fasting glucose**	Screening not recommended	Recommended for all for risk stratification and diagnosis of diabetes	Screening not recommended
**Subclinical atherosclerosis**	Consider statin therapy for asymptomatic patients at moderate risk with carotid plaque ≥0.5 mm of IMT or IMT ≥1.5 mm. Recommendations based on CCS are vague but a high CCS is a high CVD risk and a statin should be prescribed.	For intermediate risk patients consider statin therapy for patients with carotid plaque or CIMT >75th %tile for age and gender; and for a CCS >100 Agatston units.	Consider statin therapy for patients with a calculated 10 year CVD risk between 5.0% to 7.5% or even <5.0% with a CCS ≥300 Agatston units or ≥75^th^ %tile for age, gender and ethnicity.

ACC/AHA, American College of Cardiology/American Heart Association; Apo-B, apolipoprotein B; CCS,; CIMT, carotid intima-media thickness; CVD, cardiovascular disease; hsCRP, high sensitivity C-reactive protein; IMT, intima media thickness; LDL-C, low density lipoprotein cholesterol.

## Review

### Apolipoprotein B

Apolipoprotein B (apo B) is the major protein on pro-atherogenic lipoproteins (apo B-containing lipoproteins). There is one molecule of apo B in very low density lipoprotein (VLDL), VLDL remnants, low density lipoprotein (LDL) and lipoprotein (a) particles establishing levels of apo B as a reference to pro-atherogenic particles. Levels of apo B correlate well with levels of non-HDL-C, r >0.80 [8–10]. Because levels of apo B represent all pro-atherogenic particles, the replacement of fasting plasma lipids with apo B to assess CVD risk has been supported by many [11, 12]. An additional advantage of measuring apo B as compared to lipids is that fasting may not be necessary because changes in apo B100 after eating are minimally different than those measured in the fed state [13, 14]. However, although more recent analyses have found that non-HDL-C and apo B perform better than LDL-C in CVD risk prediction, both on- and off-treatment, as well as in subclinical CVD risk prediction [15], the current dogma from the Emerging Risk Factors Collaboration remains that apo B is similar to LDL-C and non-HDL-C in the prediction of CVD [16]. Moreover, when compared to total cholesterol/HDL cholesterol in primary [17] and secondary [18] CVD prevention trials, apo B was similar or weaker than the ratio, respectively, in predicting CVD events.

An important situation in which apo B may have value is in patients in whom LDL-C levels are low, for example, <100 mg/dL, and plasma triglycerides (TG) are elevated. Although levels of non-HDL-C may be helpful, apo B may provide additional information about the number of pro-atherogenic particles. It is important to realize that for any given level of non-HDL-C the 95^th^ percentage confidence intervals for apo B puts the apo B level up to two-fold different [19] and this may be especially important in the assessment and treatment of patients with hypertriglyceridemia. Because LDL-C is low, a much greater percentage of apo B is from apo B-containing particles other than LDL such as VLDL or IDL, and with a potential two-fold difference in apo B at any given level of LDL-C (<100 mg/dL), the level of apo B could be low at 65 mg/dL or high at 130 mg/dL; and thus provide markedly different levels of CVD risk. In subjects selected from 2,023 consecutive patients attending the Lipid Clinic at the Laval University Centre, 270 had mild hypertriglyceridemia and normal levels of apo B, 163 moderate hypertriglyceridemia and normal apo B, 458 mild hypertriglyceridemia with elevations in apo B, and 295 moderate hypertriglyceridemia with elevations in apo B [20]. Irrespective of levels of plasma apo B, patients with mild versus moderate hypertriglyceridemia had lower ratios of VLDL apo B/plasma apo B, a discrepancy that may be important to the CVD risk. In fact, in the Quebec Cardiovascular Study the relative risk for CVD based on apo B in patients with hypertriglyceridemia has been well documented [21]. Presently, both the Canadian Guidelines and the American College of Cardiology (ACC)/American Diabetes Association (ADA) have established goals for apo B. The Canadian Guidelines have established apo B goals of <80 mg/dL and <100 mg/dL for patients with CVD or at higher risk versus lower CVD risk [22]*.* The ACC/ADA have set goals of apo B at <80 mg/dL for patients with CVD or diabetes and one risk factor and <90 mg/dL for patients with two or more risk factors or with diabetes [23].

### Lipoprotein (a)

Lipoprotein (a) is an apo B lipoprotein that includes apolipoprotein (a) covalently bound to apo B. Plasma concentrations of lipoprotein (a) are conferred mostly by genetics that relate primarily to the size of the apo (a) protein. The size of the isoform is dependent on a variable number of kringle IV repeats in the lipoprotein (a) gene [24] and a smaller number of repeats predict a higher concentration of lipoprotein (a) [25]. Lipoprotein (a) concentrations can vary between undetectable to >200 mg/dL with a two to three fold higher level seen in populations of African descent. Plasma levels >30 mg/dL confer increased atherosclerotic risk [26]. The atherogenicity relates to multiple features of the particle including the inability of the particle to be cleared by the LDL receptor, anti-fibrinolytic properties due to the structural homology to plasminogen and competition with plasminogen for its binding site, and the particle carrying more atherogenic pro-inflammatory oxidized phospholipids [27].

The relationship between lipoprotein (a) and CVD has been well established. By 2000, there were more than 15 population-based prospective studies that reported on higher levels of lipoprotein (a) and CHD risk, with most reporting positive associations. In 2006, a study of 27,736 healthy women, of whom 12,075 indicated active hormone replacement therapy at study initiation and 15,661 did not, demonstrated that women not taking female hormones had a hazard ratio of future CVD events of 1.8 (highest lipoprotein (a) quintile versus lowest quintile, *P* <0.0001) after multifactorial risk factor adjustment [28]. For a number of years it was believed that levels >30 mg/dL were predictive of CHD events; however, more recently, a gradient relationship between higher levels of lipoprotein (a) and CVD has been evidenced. In the Reykjavik Study (n = 18,569), levels of lipoprotein (a) were measured at baseline from 2,047 patients with a non-fatal or fatal myocardial infarction (MI) versus 3,921 control participants. In addition to examining within-person fluctuations, paired samples were assessed at an interval of 12 years in 372 subjects [29]. The odds ratio for CHD, unaltered after adjustment for established risk factors was 1.60 in a comparison of extreme thirds of baseline lipoprotein (a) concentrations. Moreover, odds ratios increased in parallel with increasing levels of lipoprotein (a). In the Copenhagen Heart Study, the association of lipoprotein (a) levels with CHD was also continuous [30]. Risk rates for CHD of 1.16 and 1.13 were found after lipoprotein (a) data were adjusted for age and sex only and for lipids and other CVD risk factors, respectively, when the top and bottom lipoprotein (a) tertiles were compared. In AIM-HIGH (Atherothrombosis Intervention in Metabolic Syndrome with Low HDL/High Triglyceride and Impact on Global Health Outcomes) study, the baseline and on-study lipoprotein (a) levels were predictive of CVD events in the simvastatin plus placebo (baseline HR: 1.24, *P* = 0.002) as well as in the on-extended release niacin group (HR: 1.21, *P* = 0.017) [31]. In AIM-HIGH there was a gradient CVD risk across quartiles of lipoprotein (a). Finally, in Jupiter, baseline levels of lipoprotein (a) were not only associated with additional CVD risk, among Caucasian participants residual risk in statin-treated patients was a determinant of residual risk (adjusted HR 1.27, 95% confidence interval (CI) 1.01to 1.59; *P* = 0.04 [32].

Presently, no data exist to confirm that lowering lipoprotein (a) reduces CVD risk; however, lipoprotein (a) can be reduced by niacin, mipomersen, LDL apheresis, cholesteryl ester transfer protein inhibitors, and estrogens [33]. Of interest, estrogens may confer benefit on CVD events in post-menopausal women with the highest quintile of lipoprotein (a) [28]. A major problem with interpretation of any studies using these medications is that variably other lipoproteins are also altered favorably. The anti-sense oligonucleotide from ISIS [34] may be necessary before the independent effect of lipoprotein (a) lowering is realized.

### Homocysteine

Hyperhomocysteinemia can be as a result of deficiencies of vitamin B6, folic acid or vitamin B12 or due to a rare genetic enzyme defect. Hyperhomocysteinemia was first associated with CVD risk as it relates to the rare autosomal recessive disorder, homocystinuria. Individuals with homocystinuria have markedly elevated levels of plasma homocysteine and have a very high risk of CVD if untreated [35]. While the mechanisms are not clearly elucidated, it appears that homocysteinemia is associated with endothelial dysfunction and increased thrombosis [36]. Furthermore, observational studies, both retrospective and prospective, have shown that even moderate elevations in homocysteine, even within the normal range, are also associated with a higher risk of CVD [37, 38]. A number of clinical trials have since been published examining the effects of folic acid/B vitamin supplementation on preventing CVD events [39–45]. These studies have been done in individuals of moderate to very high risk of CVD events and, while homocysteine levels are reduced with folic acid/B vitamin supplementation, none of these studies has shown a benefit in clinical CVD outcomes. Clarke and colleagues recently published a meta-analysis of these outcome trials [46]. They included eight trials comprising a total of 37,485 individuals and found that lowering homocysteine levels by about 25% for a mean of five years was not associated with significant beneficial effects on CVD events. Specifically, no benefit was seen in major CVD events (HR 1.01, CI 0.97 to 1.05), major coronary events (HR 1.03, CI 0.97 to 1.10), stroke (HR 0.96, CI 0.87 to 1.06), or all-cause mortality (HR 1.00, CI 0.85 to 1.18) [46]. The available evidence, therefore, does not support the routine use of folic acid/B vitamin supplementation to prevent cardiovascular disease or improve overall survival, and as such there are no official recommendations for routine testing for homocysteine.

### Pro-thrombotic factors

Thrombosis is a critical process in the pathophysiology associated with acute CVD events such as acute coronary syndromes [47–49]. An unstable atherosclerotic plaque may be prone to disruption leading to platelet aggregation and acute thrombosis. Platelet activation has also been shown to play an important role in driving atherosclerosis progression as a mediator of endothelial function and inflammatory responses [48]. Furthermore, there is strong evidence supporting the benefits of antiplatelet agents, such as aspirin, in the primary and secondary therapy of CVD [50]. A recent meta-analysis found that aspirin therapy in primary prevention trials was associated with a 12% reduction in serious CVD events but no effect on stroke or vascular mortality. In secondary prevention, aspirin was associated with a more robust 18% reduction in serious CVD events [51]. Men appear to receive more benefit from aspirin in primary prevention of CHD events while women appear to receive more benefit in primary prevention of ischemic strokes [51].

It is less clear, however, whether biomarkers associated with thrombosis and platelet aggregation are helpful in clinical practice. Fibrinogen is a major coagulation protein that plays a key role in blood viscosity and platelet aggregation, and in a meta-analysis of prospective observational studies a moderately strong association has been found between fibrinogen levels and the risk of CVD [52, 53]. However, because of analytical/assay concerns and uncertainty in treatment strategies, the measurement of fibrinogen in clinical practice is not currently recommended [54]. Circulating tissue plasminogen activator (t-PA) antigen, total plasminogen inhibitor-1 (tPAI-1), D-dimer, and von Willebrand factor have also been found to be associated with increased CVD risk, but more studies are needed to assess their clinical applicability [55–57]. Furthermore, there are no known related therapeutic interventions that are available or proven successful.

### Pro-inflammatory factors

Inflammation has been known to be a critical process in the long-term progression of atherosclerosis for some time [47, 49, 58]. C-reactive protein (CRP) is an acute phase reactant that has been used as a marker of systemic inflammation in rheumatologic disorders. Retrospective and prospective studies have found that high sensitivity CRP (hsCRP) elevations are associated with acute CVD events [59]. Ridker *et al*. found that men participating in the Physicians’ Health Study who had hsCRP levels in the highest quartile had a relative risk of 2.9 for MI and 1.9 for ischemic stroke compared to those in the lowest quartile [59]. Furthermore, they found that aspirin was associated with significant reductions in the risk of MI in those with the highest hsCRP levels [59]. Ridker *et al*. also found that hsCRP was a strong predictor of CVD events in women participating in the Women’s Health Study and that hsCRP may be a stronger predictor of CVD events than LDL-C levels [60]. Furthermore, recent meta-analyses have found that hsCRP is associated with risk for CVD events and mortality [61, 62]. There also appears to be a relationship between hsCRP and LDL-C lowering. In the *PROVE IT Study*, hsCRP lowering with statin therapy was associated with reduced CVD events regardless of the LDL-C lowering [63]. In the JUPITER Study, rosuvastatin significantly decreased CVD events in patients with elevated hsCRP (>2 mg/L) and ‘normal’ LDL-C (<130 mg/dL) [64], suggesting the importance of hsCRP as a marker of CVD risk and response to statin therapy. There is little evidence, though, that lowering hsCRP levels prevents CVD events [61]. In light of these findings, the new *2013 ACC/AHA Guideline on the Assessment of Cardiovascular Risk* recommends that, based on expert opinion, measurement hsCRP may be considered as a marker of risk to inform decision making on treatment options [5]. There is evidence, though, to suggest that an anti-inflammatory agent such as methotrexate is associated with reduced CVD events in patients treated for rheumatoid arthritis [65]. As such, there are currently trials designed to examine whether anti-inflammatory agents reduce CVD risk by reducing systemic inflammation, such as the *Cardiovascular Inflammation Reduction Trial* sponsored by the National Heart, Lung, and Blood Institute and Brigham and Women’s Hospital investigating whether low dose methotrexate reduces CVD outcomes in high risk individuals, which may provide evidence for using inflammatory markers as a treatment target.

### Impaired glucose metabolism

Hyperglycemia and diabetes mellitus are clearly associated with increased CVD risk [66–68]. There is evidence, though, that mild hyperglycemia below the cutoffs for diabetes is also associated with increased CVD risk [68]. Mild hyperglycemia or ‘pre-diabetes’ can manifest as either impaired fasting glucose (IFG), impaired glucose tolerance (IGT), and/or elevated hemoglobin A1c (HbA1c). These impairments in glucose metabolism are associated with insulin resistance and other cardiometabolic risk factors, such as high blood pressure, dyslipidemia, pro-inflammatory state and pro-thrombotic state, all resulting in increased risk for CVD [69]. It is more controversial whether hyperglycemia, especially at mild, pre-diabetes levels, is a direct cause of CVD. IFG using cutoffs of 110 mg/dl (6.0 mmol/l) [70] and 100 mg/dl (5.6 mmol/l) [71] has been shown to be independently associated with increased CVD risk [72–74]. In a recent meta-analysis, Ford *et al*. found that IFG was associated with an 18% to 20% increased risk in CVD [74]. Interestingly, as has been shown in patients with ‘frank’ diabetes [66], Levitzky *et al*. found that women with IFG had a close to 1.7 to 2.2 fold increase in CHD while no effect was seen in men [73]. Others, though, have not found a sex-based difference in risk [74]. IGT has also been shown to be associated with an increased risk of CVD [74–76]. It is less clear, however, whether treating pre-diabetes improves CVD outcomes. A number of diabetes prevention studies, including the *Diabetes Prevention Program*, have been performed in individuals with IGT but none have been powered to examine CVD outcomes [77]. Thus, the modest risk of CVD seen in those with pre-diabetes may be a result of the associated comorbidities as opposed to a direct effect of the mild hyperglycemia.

### Subclinical ASCVD

Subclinical atherosclerosis is common and responsible for first CVD events including major coronary artery occlusion including sudden death in 40% to 60% of CHD patients in the United States [78]. This section will address only non-invasive techniques to assess this disease burden. Ankle-brachial index (ABI) is a cheap, easily employed method to asses for peripheral arterial disease (PAD) and as a predictor of CVD events. The ABI is the ankle systolic blood pressure divided by the brachial artery systolic blood pressure obtained while the patient is supine with a value of ≤0.9 considered abnormal. Despite its simplicity, the United States Preventive Services Task Force has determined that ‘the current evidence is insufficient to assess the balance of benefits and harms of screening for PAD and CVD risk assessment with the ABI in adults’ [79]. B-mode ultrasonography is most often utilized to assess the thickness of the arterial intimal and the medial layers (CIMT) in the common carotid artery. However, the *2013 ACC/AHA Guideline on the Assessment of Cardiovascular Risk Work Group* report judged that the evidence provided by Den Ruijter *et al*. [80] in combination with the concerns about measurement quality failed to provide sufficient rationale to recommend measuring common carotid IMT in routine clinical practice for CVD risk assessment for a first atherosclerotic cardiovascular disease (ASCVD) event [5]. Moreover, the systematic review of van den Oord *et al*. failed to demonstrate added value of carotid IMT to traditional risk models in predicting CVD events [81]. Important issues related to carotid IMT as an assessment of ASCVD risk include measurement error and standardization. The Den Ruijter *et al*. report was a meta-analysis of 14 population-based cohorts with a median follow-up of 11 years in 45,828 individuals with 4,007 MIs or strokes.

Electron-beam computed tomography (CT) measures coronary artery calcification, a process related to the lipid and apoptotic characteristics of the plaque. In 1,726, 57.7 +/- 13.3-year-old, asymptomatic individuals, an Agatston score >75th percentile was associated with a higher annualized event rate for myocardial infarction (3.6% versus 1.6%, *P* <0.05) and for cardiac death (2.2% versus 0.9%) compared with patients with scores <75th percentile [82]. Moreover, no cardiac events were observed in patients with coronary calcium scores of zero. In the Multi-Ethnic Study of Atherosclerosis (MESA), 6,814 subjects were examined over a mean follow-up period of 7.6 years to determine the area under the receiver operator characteristic (ROC) curve (AUC) and net reclassification improvement of coronary calcium in comparison to a series of additional CVD risk factors when added to the Framingham Risk Score [83]. In MESA, the coronary artery calcium was superior to other predictors of CHD/CVD, such as hsCRP, family history and ankle-brachial index, in reclassifying risk and discriminating the extent of CHD in intermediate-risk subjects. This study is particularly important because the improvement in ROC characteristics improved prediction above and beyond current multivariate prediction models.

The *2013 ACC/AHA Guideline on the Assessment of Cardiovascular Risk Work Group* notes used the systematic review by Peters *et al*. [84]. to provide evidence that assessing coronary artery calcification is likely to be the most useful of the current approaches to improving risk assessment among individuals found to be at intermediate risk after formal risk assessment [5]. Furthermore, the Work Group noted that the outcomes in the studies reviewed by Peters *et al*. [84]. and by Greenland *et al*. [85] were CHD outcomes, not hard ASCVD events that included stroke; thus, uncertainty remains regarding the contribution of assessing coronary artery calcium to estimating 10-year risk of first hard ASCVD events after formal risk assessment using the new Pooled Cohort Equations. Furthermore, issues of cost and radiation exposure related to measuring coronary calcium were discussed resulting in some uncertainty regarding potential risks of more widespread screening; thus, a Class IIb recommendation was given for individuals for whom a risk-based treatment decision is uncertain after formal risk estimation. Recent MESA data have provided additional information that not only the volumetric score but the density of the plaque need to be considered in the prediction of CVD events to follow [86]. In this analysis at any level of plaque volume, coronary artery calcium density was inversely and significantly associated with CHD and CVD risk. This result suggests that plaque remodeling by reduction in apo B-containing lipoproteins and inflammation may serve to consolidate lesions and render them more stable.

By obtaining ECG-gated images, contrast-enhanced multi-slice or multi-detector CT, also known as MDCT, provides a more sensitive method than electron beam CT to detail coronary anatomy. Moreover, diagnostic performance of coronary CT angiography has been substantially improved with the technological developments in multi-slice CT scanners which started with 4-slice and now has advanced to 320-slice capability [87]. The exact place of MDCT remains unclear but the elimination of unnecessary high dose radiation exposure is an important consideration [88]. Perhaps the best place for MDCT is as an alternative to invasive coronary angiography in asymptomatic patients who have a positive stress test [88].

High-resolution magnetic resonance imaging (MRI) with contrast may be the most promising technique for studying athero-thrombotic disease in humans [89]. Most importantly, MRI allows for the characterization of plaque composition including the lipid core, fibrosis, calcification, intra-plaque hemorrhage and importantly thrombi, and not only their presence but age, also. In asymptomatic subjects with subclinical markers of CVD and in those with no coronary calcium, coronary artery MRI has been used to detect increased vessel wall thickness [90]. Although there are limitations to its use including image resolution and imaging time, coronary MRI opens new strategies for the screening of higher risk patients for early detection and treatment as well as monitoring of lesions after therapeutic intervention.

## Conclusions

The purpose of this review was to update the science of emerging cardiometabolic risk factors that were originally discussed in the NCEP/ATPIII report of 2001 (updated in 2004). While there are more published data regarding the evidence for using these risk factors there continues to be significant debate and lack of consensus in their use as illustrated in Table 1 which summarizes more current recommendations (European, Canadian and American). Thus, the use of these biomarkers for patients at intermediate risk of a major cardiovascular event remains prudent in assisting in the identification of patients who need more aggressive LDL-C or non-HDL-C lowering therapy.

## Authors’ information

RHE is Professor of Medicine in the Divisions of Endocrinology, Metabolism and Diabetes and Cardiology and Professor of Physiology and Biophysics at the University of Colorado. RHE is the Director of the Lipid Clinic at the University of Colorado Hospital and Past-President of the American Heart Association. MC is an Associate Professor of Medicine in the Division of Endocrinology, Metabolism and Diabetes. MC is the Director of the University of Colorado Hospital LDL Apheresis Program.

## Abbreviations

ABI: ankle-brachial index
ACC: American College of Cardiology
ADA: American Diabetes Aassociation
AHA: American Heart Association
apo B: apolipoprotein B
ASCVD: atherosclerotic cardiovascular disease
ATP: adult treatment panel
AUC: area under the curve
CHD: coronary heart disease
CRP: C reactive protein
CT: computed tomography
CVD: cardiovascular disease
HbA1c: hemoglobin A1c
HDL: high density lipoprotein
HR: hazard ratio
hsCRP: high sensitivity CRP
IFG: impaired fasting glucose
IGT: impaired glucose tolerance
LDL: low density lipoprotein
MDCT: multidetector CT
MESA: Multi-ethnic Study of Atherosclerosis
MI: myocardial infarction
MRI: magnetic resonance imaging
NCEP: National Cholesterol Education Program
PAD: peripheral arterial disease
ROC: receiver operator characteristic
TG: triglycerides
t-PA: tissue plasminogen activator
tPAI-1: total plasminogen inhibitor-1
VLDL: very low density lipoprotein.
